# The Internal Secretions and the Principles of Medicine

**Published:** 1903-09

**Authors:** 


**The Internal Secretions and the Principles of Medicine.** By CHARLES E. DE M. SAJOUS, M.D. Vol. I. Pp. xxvi., 800. Philadelphia: F. A. Davis Company. 1903.

This volume, dedicated to the memory of Brown-Séquard, commences with a preface and summary of contents, from which we learn that a study of the physiology of the ductless glands leads to the conclusion that the adrenals are of overwhelming importance. The adrenal system, pictured in the frontispiece, is considered to be "directly connected with the anterior pituitary body through the solar plexus, the splanchnic nerves, and the cervico-thoracic ganglia of the sympathetic. Indeed, this diminutive organ, hardly as large as a pea, and now thought to be practically functionless, proved to be the most important organ of the body, as governing center of the adrenals, and therefore of all oxidation processes." Further, we read that "the thyroid gland, the anterior pituitary, and the adrenals were thus found to be functionally united, *i.e.* to form an autonomous system which we termed the "adrenal system." We further learn that "symptoms of infection or poisoning are all manifestations, more or less severe, of over-activity or insufficiency of the adrenal system. Indeed, the physiological action of remedies was also traced to the anterior pituitary body, the governing center of this system." We learn also that the posterior pituitary body, far from being the insignificant vestigial organ it is generally thought to be, is found to stand second in importance only to its mate, the anterior pituitary body; indeed, it proved to be the chief functional center of the nervous system, and to be the co-center with the anterior pituitary body "in sustaining the cellular metabolism of all organs."

It is clear that these views, if adopted, must modify orthodox views in physiology and pathology most profoundly, and the whole subject requires much careful study, such as is shown in the twelve chapters of the book. In the *Monthly Cyclopædia of Practical Medicine*, March, 1903, Dr. Sajous gives an epitome of his paper read before the County Medical Society, February 25th, and published in the *Philadelphia Medical Journal* of March 7th, in which he gives an outline of his views, and concludes as follows:—"In closing, we wish to state that, since the functions of the ductless glands and of the leucocytes have been known to us, medicine has appeared in a new light. The majority of abstruse problems have become normal combinations, bereft, as they now are, of theories which before were necessary to compensate for the functions of the organs we have described."

The author has been led by his marvellous conceptions to take quite original views of the minute histology of various anatomical structures, and of the physiological processes which take place in the human body.

"Imagination is often accompanied with a somewhat irregular logic," and Dr. Sajous can hardly expect that the chain of evidence offered in this volume in support of his views will stand the strain of criticism.

Before asking the reader to accept such a mass of unorthodox views it would have been well to have first placed beyond dispute a few of the more important statements which form the very foundation of the author's arguments.

In the first place it is a *sine qua non* for Dr. Sajous' theory that the essential oxygen carrier of the blood should be the plasma and not the hæmoglobin. We know of no experimental proof of this fact. Leonard Hill and Macleod have shown that plasma is capable of absorbing a certain amount of the gas, but only that amount which should be absorbed according to Dalton's law, and that after 1½ hours.

Many experiments are necessary, such as comparing the oxygen absorbing properties of plasma from adrenalectomised animals with those of plasma from normal animals.

Moore and Biede have practically proved that the *blood* in the suprarenal veins and the inferior cava contains suprarenal secretion, but they have not shown that the *plasma* from these veins will absorb more oxygen than the plasma takes from other parts.

In chapter ix. the action of adrenalin upon heart muscle is discussed, but here again there is a lack of experimental support.

Digitalis is alleged to act on the heart, not directly, but by increasing the adrenal secretion. We are not shown, however, that the drug fails to exert its specific action when administered to animals in which the suprarenal glands have been removed or their veins ligated.

We know, moreover, that digitalis, when brought into contact with curarised heart muscle, caused the latter to contract; but as regards the action of adrenalin in similar experiments, we are awaiting the results of Professor Langley's investigations.

Again, hydrocyanic acid is supposed by Sajous to act in poisonous doses by over-stimulating, and so paralysing the suprarenals; but we fail to conceive how such sudden symptoms as those of hydrocyanic poisoning can be attributed to the mere inactivity of these structures, without which an animal can live for from two to thirteen days, as shown by Langlois.

In the second place, passing to the pituitary body, we again notice a deplorable absence of experimental corroboration of the suggestions made as to its important functions.

According to Sajous, the posterior lobe of the pituitary is the centre from which all nervous energy arises; but this is not reconcilable with the fact that Vassali's and Sacchi's cats and dogs survived, by two weeks, complete destruction of the pituitary body.

Supposing, however, that the posterior pituitary were the site of origin of all nervous energy, then all impulses would of necessity have to pass through the infundibulum in order to reach the hemispheres. But Berkley, in his exhaustive researches, has failed to show "evidence that any of the nervous elements of the infundibular lobe pass beyond its limits into the infundibulum."

In fact, the only fibres which could possibly act as conducting paths from the posterior pituitary are, according to Berkley, "shrouded in a blackened aggregation of cellular masses," and their histological origin must remain a matter of some uncertainty.

A rather unsatisfactory path for such an important traffic! The author labours hard to convince his readers that such minute processes as the dendrites and axon of the neuron, and even the mitoma of the leucocyte, are but canaliculi for the circulation of oxygen-laden plasma. We should like to see this theory proved, and we shall look for great things in the second volume.

Meanwhile the book has added a new mystery to the physiology of life, but we think it more than likely that Dr. Sajous' judgment has been seriously warped by an imaginative faculty due to abnormal activity of the posterior pituitary body.

